# Outbreak of diarrhetic shellfish poisoning associated with consumption of mussels, United Kingdom, May to June 2019

**DOI:** 10.2807/1560-7917.ES.2019.24.35.1900513

**Published:** 2019-08-29

**Authors:** Nick Young, Charlotte Robin, Rachel Kwiatkowska, Charles Beck, Dominic Mellon, Penelope Edwards, Jonathan Turner, Paul Nicholls, Gavin Fearby, Debbie Lewis, Douglas Hallett, Tracy Bishop, Tracey Smith, Russell Hyndford, Lewis Coates, Andrew Turner

**Affiliations:** 1 Public Health England South West, Bristol, United Kingdom; 2 Field Service, National Infection Service, Public Health England, London, United Kingdom; 3 Population Health Sciences, Bristol Medical School, University of Bristol, Bristol, United Kingdom; 4 Public Health England South West Regional Laboratory, Bristol, United Kingdom; 5 Teignbridge District Council, Newton Abbot, United Kingdom; 6 Cornwall Port Health Authority, Cornwall Council, Falmouth, United Kingdom; 7 South Somerset District Council, Yeovil, United Kingdom; 8 Food Standards Agency, London, United Kingdom; 9 The Centre for Environment, Fisheries and Aquaculture Science (Cefas), Weymouth, United Kingdom

**Keywords:** Diarrhetic shellfish poisoning, harmful algal bloom, okadaic acid

## Abstract

We report on six cases of diarrhetic shellfish poisoning following consumption of mussels harvested in the United Kingdom. *Dinophysis* spp. in the water column was found to have increased rapidly at the production site resulting in high levels of okadaic acid-group lipophilic toxins in the flesh of consumed mussels. Clinicians and public health professionals should remain aware of algal-derived toxins being a potential cause of illness following seafood consumption.

We report on six cases of diarrhetic shellfish poisoning (DSP) following consumption of mussels in the United Kingdom (UK). The mussels contained high levels of heat-stable okadaic acid (OA)-group toxins. Here we describe the environmental and epidemiological investigation carried out in response to the outbreak.

## Outbreak identification

In June 2019 (day 0), Public Health England South West was notified by the local authority of three diners who were unwell following consumption of mussels in a restaurant 5 days earlier. The local authority had determined that the restaurant had had received a batch recall notice, also 5 days earlier, from the shellfish producer for the mussels because of elevated toxin levels but this was not seen before the mussels were served that day. On day 1, PHE South West received a report from the county neighbouring the first of gastrointestinal illness linked to mussels from the same producer. A multi-agency outbreak control team was therefore convened on day 2 and led by the PHE South West health protection team.

## Epidemiological investigation and findings

### Case finding

An alert was sent to all health protection teams across England on day 2 asking about any reported cases of gastrointestinal illness following consumption of mussels. Local authorities in areas of product distribution were informed of the identified risk by email. Persons reporting illness who were identified by local authorities as having consumed the affected mussels were asked by PHE to complete a bespoke questionnaire on exposure and clinical data.

### Case definitions

A probable case of DSP was defined as an individual with diarrhoea, three or more loose stools in 24 h, or vomiting or abdominal cramps or nausea, with date of onset from 7 days before to 1 day after notification of the outbreak, and time of onset 30 min to 24 h following consumption of mussels harvested from the affected site. Confirmed cases were as probable, but with an absence of pathogens in a stool sample that would otherwise explain illness.

### Results

Thirteen individuals reported to have been unwell after consumption of mussels were contacted. Completed questionnaires were received from seven individuals, of which three were confirmed, and three probable cases. The cases ate at four separate venues. One respondent did not meet the case definition as symptom onset was more than 24 h following consumption.

The epidemic curve for the outbreak is shown in Figure.

**Figure:**
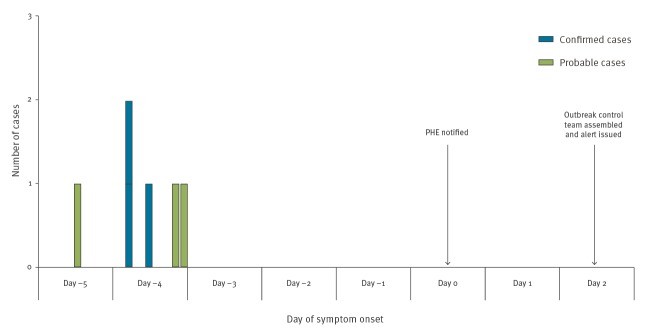
Diarrhetic shellfish poisoning cases by day of symptom onset and measures taken, United Kingdom, May–June 2019 (n = 6)

PHE: Public Health England.

The mean age of cases was 59 years (range: 37–76 years); three were male and three were female. All cases reported eating steamed mussels. Five cases ate mussels as a main course and one as a starter. Reported portion sizes ranged from 11 to 50 mussels.

The symptoms reported by cases are summarised in Table 1.

**Table 1:** Symptoms reported by diarrhetic shellfish poisoning cases, United Kingdom, May–June 2019 (n = 6)

Symptoms	Number of cases
Diarrhoea	6
Nausea	6
Abdominal pain	6
Chills	4
Fever	1
Vomiting	2
Other^a^	2

^a^ Other includes lethargy, dizziness/fainting, heart palpitations and memory loss.

The mean incubation period was 11.3 h (range: 8–17 h) and the median duration of symptoms was 2.5 days. One case sought medical advice. There were no hospitalisations.

Faecal samples were tested from the three confirmed cases and the respondent not meeting the case definition. Samples were collected at a median of 3 days (range: 1–9 days) after resolution of symptoms. All four stool samples were microscopy and culture negative for *Salmonella* spp., *Shigella* spp., *Escherichia coli* O157, *Campylobacter* spp., *Vibrio* spp., *Bacillus cereus* and *Clostridium perfringens*. Anaerobic cultures were negative. Two stool samples yielded scanty growth of *Staphylococcus aureus* that was not considered commensurate with a root cause of food poisoning. Ova, cysts and parasites were not seen on concentration. Enzyme immunoassay was negative for *Cryptosporidium* and *Giardia*. Viral PCR testing was negative for rotavirus, sapovirus, astrovirus, adenovirus and norovirus.

## Environmental investigation and findings

The mussels were produced in an offshore marine area. A routine shellfish monitoring programme is in place throughout England and Wales, including at the affected site. As a part of this programme, the water column is sampled every 2 weeks from April to September and cell counts of potentially harmful algal species are measured. Shellfish flesh samples are also tested for the presence of selected European Union (EU)-regulated biotoxins every 4 weeks during April to September each year unless phytoplankton counts and/or shellfish toxins are quantified above specified warning limits that require further precautions, including re-testing and closure.

Lipophilic toxin determination, including that for OA-group toxins, is routinely carried out using the method specified in in the EU-Harmonised Standard Operating Procedure for determination of lipophilic marine biotoxins in molluscs by LC-MS/MS [1]. Additional flesh and water samples were taken in advance of the planned sampling date following a report to the local authority from a local fisherman of a red-coloured algal bloom six miles offshore from the production site.

The local authority determined the source of the mussels by questioning venues linked to reports of illness. Subsequently, the shellfish producer provided the outbreak control team with a complete list of all businesses who had received the affected mussels. Mussels from the site were harvested daily from 9 to 5 days before notification of the outbreak for commercial sale. The mussels were not tested by the producer for the presence of toxins. A large volume of mussels was distributed to seafood wholesalers, restaurants and pubs, and subject to the recall notice distributed by the producer 5 days before reports of illness to PHE. A limited number of businesses not linked to any known cases, including wholesalers, retailers, restaurants and pubs, responded to the recall stating they had sold some of the affected produce. No produce was found to still be in circulation at the time of the outbreak response.

Water column and shellfish flesh sampling results are summarised in Table 2. Measured densities of *Dinophysis* spp. in the water column increased rapidly from being undetectable 16 days before outbreak notification to 1,600 cells per litre 7 days before, coinciding with the time of harvesting of the affected batch and exceeding the England, Wales and Northern Ireland Food Standards Agency trigger level of 100 cells per litre. The level of total OA-group lipophilic toxins in mussel flesh was 338 µg OA equivalents (eq) per kg, following application of measurement uncertainty, 7 days before outbreak notification. This exceeded the maximum permitted limit (MPL) of 160 µg OA eq per kg defined by European Commission (EC) regulation 853/2004 [2]. Toxin concentrations quantified showed that an average of 94% of the OA-group toxins present in the mussels consisted of OA itself, with the remainder being dinophysistoxin 2 (DTX2).

**Table 2:** Summary of flesh okadaic acid-group toxicity in mussels and Dinophyceae cell counts in water from the affected site, United Kingdom, April–June 2019

Date of collection^a^	Total OA-group toxicity in mussel flesh (µg OA eq/kg)	Dinophyceae cell counts (cells/L)
Day −55	ND	40
Day −34	34	ND
Day −16	Not sampled	ND
Day −7	338	1,600
Day −1	499	200
Day 6	270	Not sampled
Day 12	121	Not sampled
Day 19	106	ND

Eq: equivalents; OA: okadaic acid; ND: not detected.

^a^ In relation to day 0, when Public Health England South West was notified of three diners who were unwell following consumption of mussels in a restaurant.

Water column sampling 7 days before outbreak notification did not detect other harmful algal species apart from *Pseudo-nitzschia* spp., the causative diatom for domoic acid responsible for amnesic shellfish poisoning, at 1,320 cells per litre. This is below the trigger level of 150,000 cells per litre for this species.

Routine shellfish sampling at the same site during the same time period did not detect paralytic shellfish poisoning toxins. Trace levels of yessotoxins were detected, but along with traces of azaspiracids, they were well below regulatory levels. Amnesic shellfish poisoning toxins were below the limit of quantitation (LOQ).

## Control measures

In response to the elevated toxin levels quantified and reported 5 days before outbreak notification, the shellfish bed was immediately closed for harvesting as per standard practice in England. The Food Standards Agency urgently contacted local authorities of places where the affected product had been distributed to ensure that wholesalers and venues had acted upon the recall. Venues were asked whether any product had been frozen, for example in the form of stock, as this would not deactivate the toxin, but there was no evidence this had been done.

## Discussion

We report on six cases of DSP associated with consumption of mussels harvested in the South West of England. Without an available validated test for relevant toxins in human samples, the diagnosis was made based on characteristic clinical symptoms, including diarrhoea, abdominal pain, nausea and fever/chills, elevated levels of OA-group toxins in the flesh of mussels from the same batch as those consumed, the absence of faecal pathogens in stool of cases and epidemiological evidence of exposure to the produce.

DSP occurs following consumption of seafood containing high levels of the heat-stable OA-group toxins produced by dinoflagellates including *Dinophysis* spp., and is characterised by a rapid-onset of self-limiting gastrointestinal illness [3,4]. Recognised outbreaks of DSP are rare. Seventy cases were identified in 2013 following consumption of mussels harvested around the Shetland Islands [5] and 49 cases were identified in 1998 following consumption of UK-harvested mussels in London [6]. Outbreaks have been recorded in recent years in China, the United States, France and Canada [4,7-9].

The lowest-observed-adverse-effect level of OA is 45 to 50 µg OA eq per person [4,10]. In our study, an average main course portion of mussels (500 g in shell) would provide 41 µg OA eq., using a flesh weight yield of 24% [11]. This level of exposure is consistent with DSP as the cause of illness considering variability in portion sizes, flesh yield, body weight and toxin levels at the production site. Individual mussel sizes served were unavailable but would likely vary. Therefore, overall estimated portion weight was used to calculate the exposure dose. A limitation is that body weight (bw) was not recorded for cases and because of this, OA eq per kg bw could not be calculated.

A shellfish biotoxin programme monitoring the occurrence of harmful algal blooms and toxins in classified shellfish production areas in the UK, alongside food business operator checks, remains a robust system to protect population health. Nonetheless, a rapid increase in concentrations of *Dinophysis spp.* cells within the waters of the production site may have contributed to the outbreak, in tandem with shellfish harvesting occurring before official control results were reported and site closure. Whyte et al. (2014) demonstrated that a similar rapid increase in *Dinophysis* levels, resulting from a change in prevailing wind direction, occurred in the 2013 Shetland Islands origin outbreak [5]. Transdisciplinary research is required to predict future risk and inform monitoring, particularly given likely changes in the distribution of potentially-toxic species particularly if temperature of ocean water increases [12]. Our investigation suggested that affected produce may have been sold by restaurants and pubs with no known linked cases. Given that DSP is a self-limiting illness that may be under-reported by cases and has low awareness among clinicians, the actual number of persons affected in this outbreak is likely to be higher [13].

This outbreak highlights that clinicians and public health professionals should be aware of algal-derived toxins as a potential cause of illness following seafood consumption, and that the need for effective end-product testing of shellfish to ensure food safety remains.
